# 5,7,7,12,14,14-Hexamethyl-4,8-diaza-1,11-diazo­niocyclotetra­deca-4,11-diene diiodide dihydrate

**DOI:** 10.1107/S1600536811005848

**Published:** 2011-02-23

**Authors:** Alan R. Kennedy, Samwel T. Lutta, Catriona A. Morrison, Maurice O. Okoth, Daniel M. Orang’o

**Affiliations:** a Department of Pure & Applied Chemistry, University of Strathclyde, 295 Cathedral Street, Glasgow G1 1XL, Scotland; b Department of Chemistry and Biochemistry, Moi University, PO Box 1125-30100, Eldoret, Kenya

## Abstract

The asymmetric unit of the title compound, C_16_H_34_N_4_^2+^·2I^−^·2H_2_O, contains one half-cation, one iodide anion and one water mol­ecule. The cation has crystallographically imposed centrosymmetric symmetry. Despite some differences in the unit-cell dimensions, packing analysis on a cluster of 15 cations and a comparison of the hydrogen bonding suggests that this compound is isostructural with its bromide analogue. Inter­molecular hydrogen bonding forms eight-membered [H—O—H⋯I]_2_ and [H—N—H⋯I]_2_ rings and creates a sheet structure.

## Related literature

For the preparation and structure of the equivalent bromide salt, see: Rohovec *et al.* (1999 ▶). For the structure of the perchlorate salt, see: Bi *et al.* (2008 ▶). For structures of representative transition metal complexes, see: Bieńko *et al.* (2007 ▶); Yang (2005 ▶); Ballester *et al.* (2000 ▶); Endicott *et al.* (1981 ▶); Wester *et al.* (1977 ▶); Goedken *et al.* (1973 ▶). Macrocyclic metal complexes have been studied extensively owing to their similarity to metallobiomolecules, and in order to further understanding of biological mechanisms, see: Merrell *et al.* (1977 ▶). The packing analysis was performed with *Mercury* (Macrae *et al.*, 2008 ▶). 
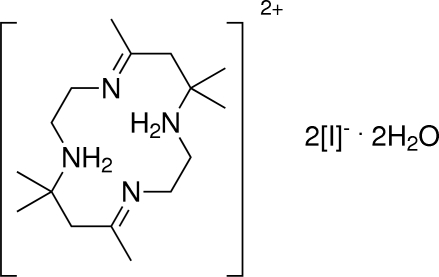


## Experimental

### 

#### Crystal data

C_16_H_34_N_4_^2+^·2I^−^·2H_2_O
*M* *_r_* = 572.30
Triclinic, 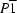

*a* = 8.4098 (3) Å
*b* = 8.7252 (2) Å
*c* = 8.7724 (3) Å
α = 74.673 (2)°
β = 66.267 (1)°
γ = 75.809 (2)°
*V* = 561.24 (3) Å^3^
*Z* = 1
Mo *K*α radiation
μ = 2.82 mm^−1^
*T* = 120 K
0.20 × 0.14 × 0.10 mm

#### Data collection

Bruker–Nonius Roper CCD diffractometer
Absorption correction: multi-scan (*SADABS*; Sheldrick, 2007 ▶) *T*_min_ = 0.673, *T*_max_ = 0.746
12010 measured reflections
2563 independent reflections
2478 reflections with *I* > 2σ(*I*)
*R*_int_ = 0.030

#### Refinement

*R*[*F* ^2^ > 2σ(*F* ^2^)] = 0.019
*wR*(*F* ^2^) = 0.046
*S* = 1.18
2563 reflections
127 parameters
3 restraints
H atoms treated by a mixture of independent and constrained refinement
Δρ_max_ = 0.99 e Å^−3^
Δρ_min_ = −0.75 e Å^−3^

### 

Data collection: *COLLECT* (Hooft, 1988 ▶); cell refinement: *DENZO* (Otwinowski & Minor, 1997 ▶) and *COLLECT* ; data reduction: *DENZO* and *COLLECT*; program(s) used to solve structure: *SIR2004* (Burla *et al.*, 2005) ▶; program(s) used to refine structure: *SHELXL97* (Sheldrick, 2008 ▶); molecular graphics: *ORTEP-3* (Farrugia, 1997 ▶); software used to prepare material for publication: *SHELXL97*.

## Supplementary Material

Crystal structure: contains datablocks global, I. DOI: 10.1107/S1600536811005848/rk2263sup1.cif

Structure factors: contains datablocks I. DOI: 10.1107/S1600536811005848/rk2263Isup2.hkl

Additional supplementary materials:  crystallographic information; 3D view; checkCIF report

## Figures and Tables

**Table 1:** Hydrogen-bond geometry (Å, °)

*D*—H⋯*A*	*D*—H	H⋯*A*	*D*⋯*A*	*D*—H⋯*A*
N1—H1*N*⋯N2^i^	0.89 (3)	2.04 (3)	2.744 (2)	136 (2)
O1*W*—H1*W*⋯I1	0.88 (2)	2.71 (2)	3.5753 (18)	171 (3)
O1*W*—H2*W*⋯I1^ii^	0.87 (2)	2.68 (2)	3.5494 (17)	176 (3)
N1—H2*N*⋯I1^ii^	0.81 (3)	3.23 (3)	3.6895 (17)	119 (2)
N1—H2*N*⋯I1^iii^	0.81 (3)	2.99 (3)	3.7110 (18)	149 (2)

Symmetry codes: (i) 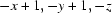; (ii) 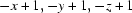; (iii) 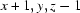.

**Fig. 1.**
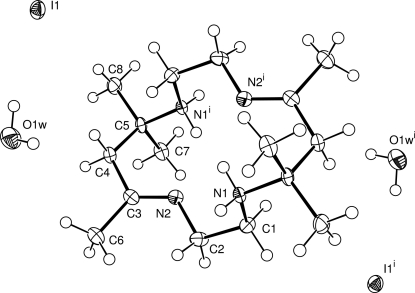
The molecular structure of the macrocyclic dication with atom numbering scheme. Displacement ellipsoids are drawn at 50% probability level H atoms are presented as a small sphertes of arbitrary radius. Symmetry code: (i) 1-x, 1-y, -z.

**Fig. 2.**
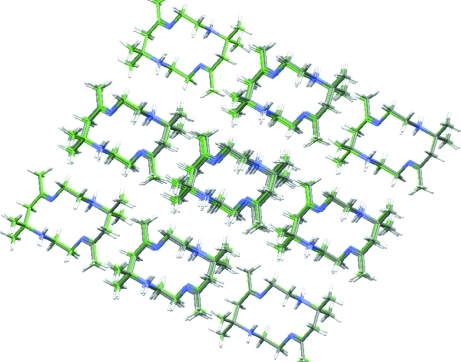
Overlaid packing diagram, showing cations from the iodide structure in green and those from the bromide structure in blue.

## supplementary crystallographic information

### Comment

Macrocyclic metal complexes have been studied extensively owing to their similarity to metallobiomolecules, and in order to further understanding of biological mechanisms (Merril *et al.*, 1977). The title molecule, 5,7,7,12,14, 14–hexamethyl–4,8–diaza–1,11–diazoniocyclo–4,11–tetradecadiene diiodide dihydrate, I, is the hydroiodide salt of an imine based ligand that has been used extensively to form complexes with the later first row transition metals. These are typically cobalt, nickel and copper complexes (see, for example, Endicott *et al.*, 1981; Ballester *et al.*, 2000; Bieńko *et al.*, 2007) but structural examples with iron, zinc and even chromium are also known (Goedken *et al.*, 1973; Yang, 2005; Wester *et al.*, 1977). The structures of the free base and of the bromide and perchlorate salts have also been reported (Rohovec *et al.*, 1999; Bi *et al.*, 2008).

The macrocyclic dication has crystallographically imposed centrosymetric symmetry, *Z*' = 1/2, with protonation at the amine N–atoms rather than at the imine groups (Fig. 1). The unit–cell parameters are somewhat similar to those of the bromide analogue (Rohovec *et al.*, 1999) measured at room temperature. However, there is a difference in that the most acute angle subtends the longest and shortest cell axes in I, but subtends the shortest and middle length cell axes in the iodide salt. To check if this was a structurally significant variation the "crystal packing similarity" module of Mercury CSD 2.3 was used (Macrae *et al.*, 2008). This analysis of the largest molecular component in the array (here the macrocyclic cation) showed that a molecular cluster of fifteen cations from each salt matched to within distance and torsion angle variations of 20%. Thus the two structures are isostructural, see overlay in Fig. 2.

Classical intramolecular N—H···N hydrogen–bonding joins the amine and imine N–atoms across the macrocycle. There are also four independent intermolecular hydrogen–bonds. All involve iodide as the acceptor with both water H–atoms acting as donors and atom H2N acting as a donor in two seperate interactions, see Table 1. Eight membered [H—O—H···I]_2_ and [H—N—H···I]_2_ rings support a two dimensional sheet structure propagated largely through N—H···I interactions. This is again similar to the bromide structure and so their isostructural nature is confirmed.

### Experimental

A 0.2 mol (13.2 mL) sample of ethylenediamine (*ED*) was put into 10 ml absolute ethanol and cooled in an ice bath for about 10 minutes. A 0.2 mol (36.2 ml of 55%) sample of hydroiodic acid was slowly added to the cool *ED* solution. Care was taken not to let the solution to boil over. After the addition of HI, 30 mL of acetone was added (an excess of 0.4 mL was required) and the solution allowed to cool in an ice bath overnight. The colourless crystalline material was filtered from solution. It was washed in absolute *Et*OH and dried in air for 30 minutes (yield 6.221 g).

### Refinement

The position of the nitrogen–bound H atoms were refined freely, but the positions of the water H atoms were restrained such that O—H and H···H distances approximated 0.88Å and 1.33Å respectively with *U*_iso_(H) set to 1.5 *U*_eq_(O). All other H atoms were placed in calculated positions and refined in riding modes with C—H = 0.98Å or 0.99Å for the CH_3_ and CH_2_ groups respectively. The *U*_iso_(H) values were set to 1.5 or 1.2 times *U*_eq_ of their parent C atoms for the CH_3_ and CH_2_ groups respectively.

### Crystal data

**Table:**

C_16_H_34_N_4_^2+^·2I^−^·2H_2_O	*Z* = 1
*M**_r_* = 572.30	*F*(000) = 284
Triclinic, *P*1	*D*_x_ = 1.693 Mg m^−^^3^
Hall symbol: -P 1	Mo *K*α radiation, λ = 0.71073 Å
*a* = 8.4098 (3) Å	Cell parameters from 10421 reflections
*b* = 8.7252 (2) Å	θ = 2.9–27.5°
*c* = 8.7724 (3) Å	µ = 2.82 mm^−^^1^
α = 74.673 (2)°	*T* = 120 K
β = 66.267 (1)°	Block, colourless
γ = 75.809 (2)°	0.20 × 0.14 × 0.10 mm
*V* = 561.24 (3) Å^3^	

### Data collection

**Table:**

Bruker–Nonius Roper CCD diffractometer	2563 independent reflections
Radiation source: Bruker–Nonius FR591 rotating anode	2478 reflections with *I* > 2σ(*I*)
graphite	*R*_int_ = 0.030
Detector resolution: 9.091 pixels mm^-1^	θ_max_ = 27.5°, θ_min_ = 3.2°
φ and ω scans	*h* = −10→10
Absorption correction: multi-scan (*SADABS*; Sheldrick, 2007)	*k* = −11→11
*T*_min_ = 0.673, *T*_max_ = 0.746	*l* = −11→11
12010 measured reflections	

### Refinement

**Table:**

Refinement on *F*^2^	Secondary atom site location: difference Fourier map
Least-squares matrix: full	Hydrogen site location: inferred from neighbouring sites
*R*[*F*^2^ > 2σ(*F*^2^)] = 0.019	H atoms treated by a mixture of independent and constrained refinement
*wR*(*F*^2^) = 0.046	*w* = 1/[σ^2^(*F*_o_^2^) + (0.0124*P*)^2^ + 0.33*P*] where *P* = (*F*_o_^2^ + 2*F*_c_^2^)/3
*S* = 1.18	(Δ/σ)_max_ = 0.001
2563 reflections	Δρ_max_ = 0.99 e Å^−^^3^
127 parameters	Δρ_min_ = −0.75 e Å^−^^3^
3 restraints	Extinction correction: *SHELXL97* (Sheldrick, 2008), Fc^*^=kFc[1+0.001xFc^2^λ^3^/sin(2θ)]^-1/4^
Primary atom site location: structure-invariant direct methods	Extinction coefficient: 0.0243 (13)

### Special details

**Table:**

Experimental. Southampton NCS collection 2010src0073
Geometry. All s.u.'s (except the s.u. in the dihedral angle between two l.s. planes) are estimated using the full covariance matrix. The cell s.u.'s are taken into account individually in the estimation of s.u.'s in distances, angles and torsion angles; correlations between s.u.'s in cell parameters are only used when they are defined by crystal symmetry. An approximate (isotropic) treatment of cell s.u.'s is used for estimating s.u.'s involving l.s. planes.
Refinement. Refinement of *F*^2^ against ALL reflections. The weighted *R*–factor *wR* and goodness of fit *S* are based on *F*^2^, conventional *R*–factors *R* are based on *F*, with *F* set to zero for negative *F*^2^. The threshold expression of *F*^2^ > σ(*F*^2^) is used only for calculating *R*–factors(gt) *etc*. and is not relevant to the choice of reflections for refinement. *R*–factors based on *F*^2^ are statistically about twice as large as those based on *F*, and *R*–factors based on ALL data will be even larger.

### Fractional atomic coordinates and isotropic or equivalent isotropic displacement parameters (Å^2^)

**Table:**

	*x*	*y*	*z*	*U*_iso_*/*U*_eq_	
I1	0.214555 (16)	0.460869 (15)	0.757490 (15)	0.02330 (8)	
O1W	0.6274 (2)	0.21795 (19)	0.5780 (2)	0.0336 (4)	
H1W	0.525 (3)	0.270 (3)	0.633 (3)	0.050*	
H2W	0.671 (3)	0.295 (3)	0.496 (3)	0.050*	
N1	0.7414 (2)	0.60362 (18)	−0.1726 (2)	0.0139 (3)	
N2	0.6077 (2)	0.29338 (18)	0.0038 (2)	0.0165 (3)	
C1	0.7419 (3)	0.4771 (2)	−0.2576 (2)	0.0185 (4)	
H1A	0.8389	0.4822	−0.3694	0.022*	
H1B	0.6296	0.4954	−0.2762	0.022*	
C2	0.7653 (3)	0.3134 (2)	−0.1486 (3)	0.0209 (4)	
H2A	0.7880	0.2286	−0.2140	0.025*	
H2B	0.8680	0.3019	−0.1159	0.025*	
C3	0.6167 (3)	0.1933 (2)	0.1357 (3)	0.0174 (4)	
C4	0.4514 (3)	0.1743 (2)	0.2906 (2)	0.0182 (4)	
H4A	0.4494	0.2381	0.3696	0.022*	
H4B	0.4604	0.0599	0.3474	0.022*	
C5	0.2753 (3)	0.2226 (2)	0.2659 (2)	0.0158 (4)	
C6	0.7788 (3)	0.0876 (3)	0.1592 (3)	0.0275 (5)	
H6A	0.7804	−0.0226	0.1507	0.041*	
H6B	0.7780	0.0881	0.2711	0.041*	
H6C	0.8836	0.1283	0.0712	0.041*	
C7	0.2617 (3)	0.1223 (2)	0.1546 (3)	0.0214 (4)	
H7A	0.1452	0.1527	0.1456	0.032*	
H7B	0.2788	0.0079	0.2052	0.032*	
H7C	0.3523	0.1417	0.0414	0.032*	
C8	0.1260 (3)	0.2045 (2)	0.4379 (3)	0.0235 (4)	
H8A	0.1421	0.2616	0.5118	0.035*	
H8B	0.1266	0.0902	0.4895	0.035*	
H8C	0.0134	0.2502	0.4230	0.035*	
H1N	0.649 (3)	0.599 (3)	−0.076 (3)	0.020 (6)*	
H2N	0.829 (4)	0.584 (3)	−0.149 (3)	0.029 (7)*	

### Atomic displacement parameters (Å^2^)

**Table:**

	*U*^11^	*U*^22^	*U*^33^	*U*^12^	*U*^13^	*U*^23^
I1	0.01952 (10)	0.03155 (11)	0.01896 (10)	−0.00480 (6)	−0.00879 (7)	−0.00124 (6)
O1W	0.0384 (10)	0.0238 (8)	0.0371 (10)	−0.0029 (7)	−0.0157 (8)	−0.0017 (7)
N1	0.0139 (8)	0.0133 (7)	0.0155 (8)	−0.0038 (6)	−0.0075 (7)	0.0007 (6)
N2	0.0160 (8)	0.0143 (7)	0.0190 (8)	−0.0048 (6)	−0.0052 (7)	−0.0025 (6)
C1	0.0213 (10)	0.0168 (9)	0.0173 (10)	−0.0052 (7)	−0.0053 (8)	−0.0036 (7)
C2	0.0180 (10)	0.0141 (9)	0.0256 (11)	−0.0041 (7)	−0.0017 (8)	−0.0040 (8)
C3	0.0173 (9)	0.0153 (9)	0.0240 (10)	−0.0027 (7)	−0.0105 (8)	−0.0055 (7)
C4	0.0197 (10)	0.0172 (9)	0.0185 (9)	−0.0036 (7)	−0.0101 (8)	0.0011 (7)
C5	0.0190 (10)	0.0118 (8)	0.0171 (9)	−0.0053 (7)	−0.0092 (8)	0.0031 (7)
C6	0.0221 (11)	0.0293 (11)	0.0303 (12)	0.0028 (8)	−0.0137 (9)	−0.0038 (9)
C7	0.0261 (11)	0.0155 (9)	0.0284 (11)	−0.0071 (8)	−0.0154 (9)	−0.0012 (8)
C8	0.0212 (11)	0.0219 (10)	0.0218 (11)	−0.0070 (8)	−0.0055 (8)	0.0044 (8)

### Geometric parameters (Å, °)

**Table:**

O1W—H1W	0.877 (17)	C4—C5	1.524 (3)
O1W—H2W	0.873 (17)	C4—H4A	0.9900
N1—C1	1.485 (2)	C4—H4B	0.9900
N1—C5^i^	1.524 (2)	C5—N1^i^	1.524 (2)
N1—H1N	0.89 (3)	C5—C8	1.524 (3)
N1—H2N	0.81 (3)	C5—C7	1.524 (3)
N2—C3	1.269 (3)	C6—H6A	0.9800
N2—C2	1.462 (2)	C6—H6B	0.9800
C1—C2	1.512 (3)	C6—H6C	0.9800
C1—H1A	0.9900	C7—H7A	0.9800
C1—H1B	0.9900	C7—H7B	0.9800
C2—H2A	0.9900	C7—H7C	0.9800
C2—H2B	0.9900	C8—H8A	0.9800
C3—C6	1.504 (3)	C8—H8B	0.9800
C3—C4	1.510 (3)	C8—H8C	0.9800
			
H1W—O1W—H2W	101 (2)	C5—C4—H4B	107.8
C1—N1—C5^i^	117.45 (15)	H4A—C4—H4B	107.1
C1—N1—H1N	107.0 (15)	N1^i^—C5—C4	109.64 (15)
C5^i^—N1—H1N	105.9 (15)	N1^i^—C5—C8	109.95 (16)
C1—N1—H2N	109.8 (18)	C4—C5—C8	109.65 (16)
C5^i^—N1—H2N	108.5 (18)	N1^i^—C5—C7	105.81 (15)
H1N—N1—H2N	108 (2)	C4—C5—C7	111.51 (16)
C3—N2—C2	120.48 (17)	C8—C5—C7	110.21 (16)
N1—C1—C2	109.64 (16)	C3—C6—H6A	109.5
N1—C1—H1A	109.7	C3—C6—H6B	109.5
C2—C1—H1A	109.7	H6A—C6—H6B	109.5
N1—C1—H1B	109.7	C3—C6—H6C	109.5
C2—C1—H1B	109.7	H6A—C6—H6C	109.5
H1A—C1—H1B	108.2	H6B—C6—H6C	109.5
N2—C2—C1	110.39 (16)	C5—C7—H7A	109.5
N2—C2—H2A	109.6	C5—C7—H7B	109.5
C1—C2—H2A	109.6	H7A—C7—H7B	109.5
N2—C2—H2B	109.6	C5—C7—H7C	109.5
C1—C2—H2B	109.6	H7A—C7—H7C	109.5
H2A—C2—H2B	108.1	H7B—C7—H7C	109.5
N2—C3—C6	127.05 (19)	C5—C8—H8A	109.5
N2—C3—C4	119.23 (17)	C5—C8—H8B	109.5
C6—C3—C4	113.71 (17)	H8A—C8—H8B	109.5
C3—C4—C5	118.10 (16)	C5—C8—H8C	109.5
C3—C4—H4A	107.8	H8A—C8—H8C	109.5
C5—C4—H4A	107.8	H8B—C8—H8C	109.5
C3—C4—H4B	107.8		
			
C5^i^—N1—C1—C2	178.16 (16)	N2—C3—C4—C5	23.3 (3)
C3—N2—C2—C1	−156.80 (17)	C6—C3—C4—C5	−157.41 (17)
N1—C1—C2—N2	69.8 (2)	C3—C4—C5—N1^i^	−55.5 (2)
C2—N2—C3—C6	1.3 (3)	C3—C4—C5—C8	−176.29 (16)
C2—N2—C3—C4	−179.47 (16)	C3—C4—C5—C7	61.4 (2)

Symmetry codes: (i) −*x*+1, −*y*+1, −*z*.

### Hydrogen-bond geometry (Å, °)

**Table:**

*D*—H···*A*	*D*—H	H···*A*	*D*···*A*	*D*—H···*A*
N1—H1N···N2^i^	0.89 (3)	2.04 (3)	2.744 (2)	136 (2)
O1W—H1W···I1	0.88 (2)	2.71 (2)	3.5753 (18)	171 (3)
O1W—H2W···I1^ii^	0.87 (2)	2.68 (2)	3.5494 (17)	176 (3)
N1—H2N···I1^ii^	0.81 (3)	3.23 (3)	3.6895 (17)	119 (2)
N1—H2N···I1^iii^	0.81 (3)	2.99 (3)	3.7110 (18)	149 (2)

Symmetry codes: (i) −*x*+1, −*y*+1, −*z*; (ii) −*x*+1, −*y*+1, −*z*+1; (iii) *x*+1, *y*, *z*−1.
